# Supplementary material for: A human lung tumor microenvironment interactome identifies clinically relevant cell-type cross-talk
Source: Genome Biol. 2020 May 7;21:107. doi: 10.1186/s13059-020-02019-x (PMC7206807; doi:10.1186/s13059-020-02019-x)
Supplement: Supplementary file 14 — Supplementary figures. [file 13059_2020_2019_MOESM14_ESM.docx]

**SUPPLEMENTARY TABLE LEGENDS AND SUPPLEMENTARY FIGURES**

**Figure S1: Transcriptional output across feature types and tissues**

Summary of the number of protein coding genes and lincRNAs that are expressed at specific TPM thresholds in 1, 2, 3, or 4 sorted populations.

**Figure S2**

Check of sample identities by comparing SNPs. All pairwise comparisons were done using *Bammatch*. Similarity was computed as the proportion of shared SNPs (upper right of figure). Bottom left panel shows sample similarity vs number of SNPs compared. Red are samples from the same tumor; yellow from different tumors.


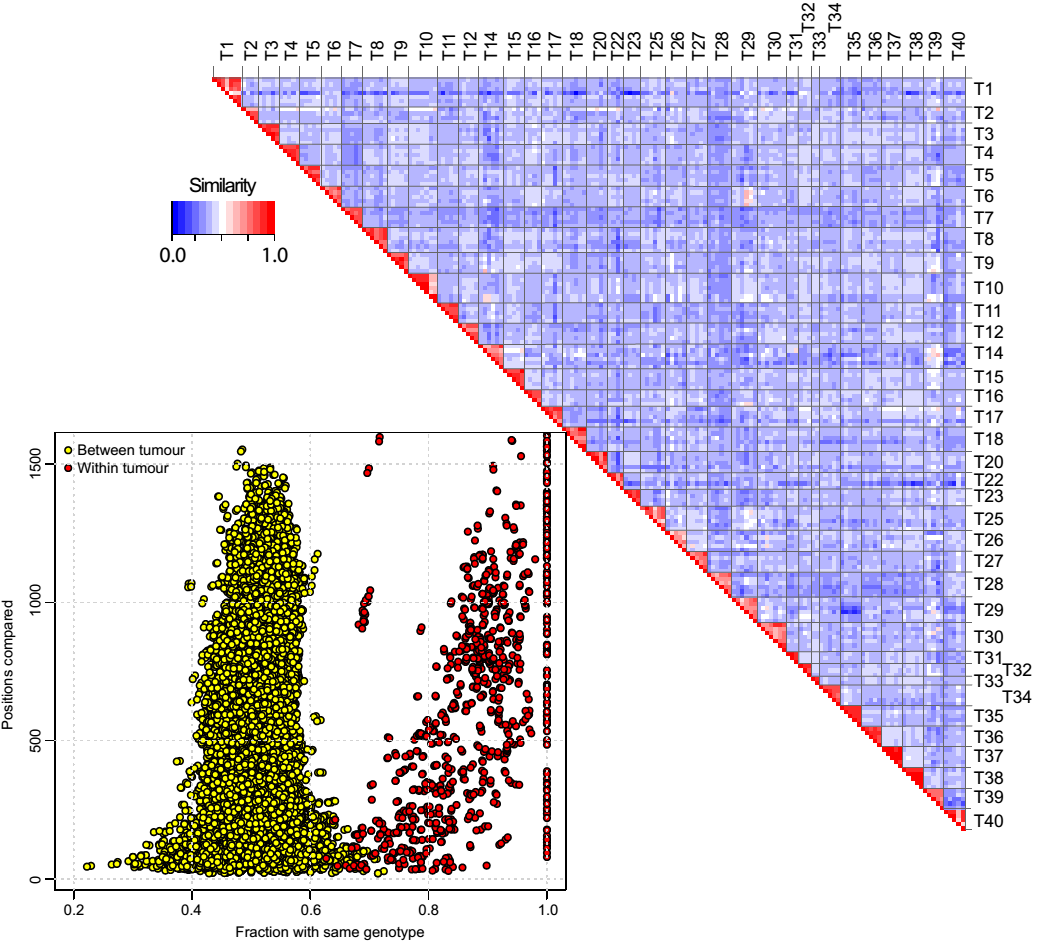


**Figure S3**

Example of effects of batch correction on two representative samples; one with good initial concordance and one with poor concordance. The blue density plot represent all genes quantified; dots are outliers.


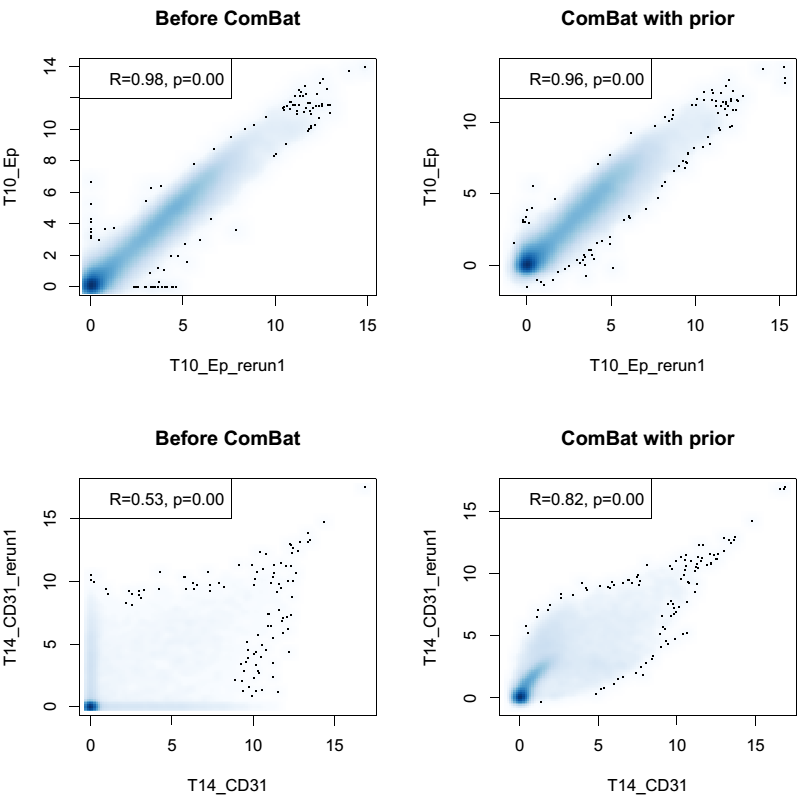


**Figure S4 – CIBERSORT signature matrix**

Depiction of CIBERSORT signature matrix used for inferring proportions of cell types in bulk tumors. Rows represent genes (n=652); see Supplementary Table 4 for the actual genes and their average expression in each cell type class.

**Figure S5 – CIBERSORT deconvolution of immune populations in bulk RNA-seq and PRECOG microarray samples.**

Comparison of immune proportions inferred in lung adenocarinoma and SCC by CIBERSORT from bulk RNA-seq (this study) and from microarrays (PRECOG)

**Figure S6: Full Western blot ladders for alpha-tubulin, Gremlin-1, KDR, and phospho-KDR (related to Figure 3j)**

| 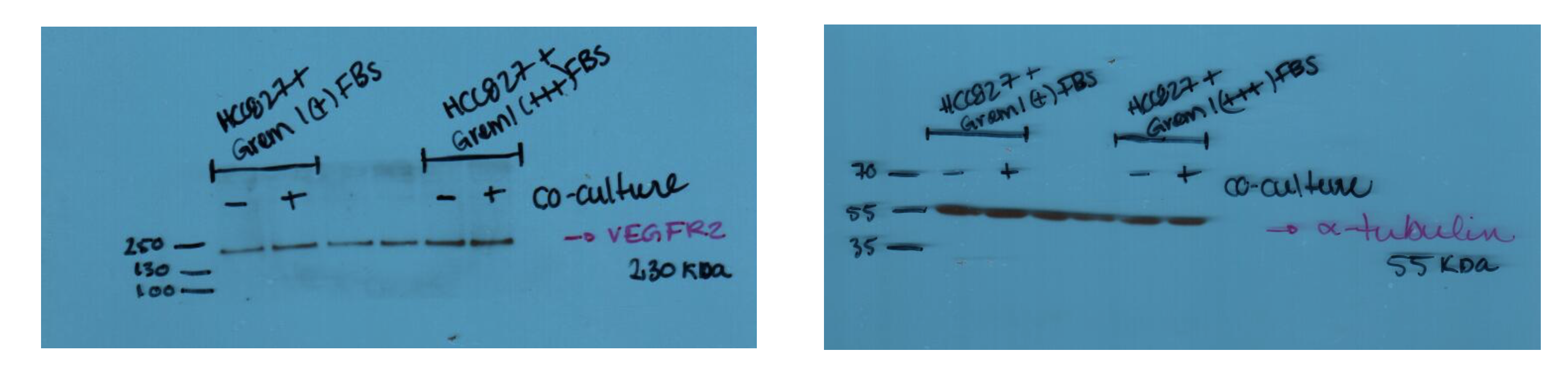 |
| --- |
| 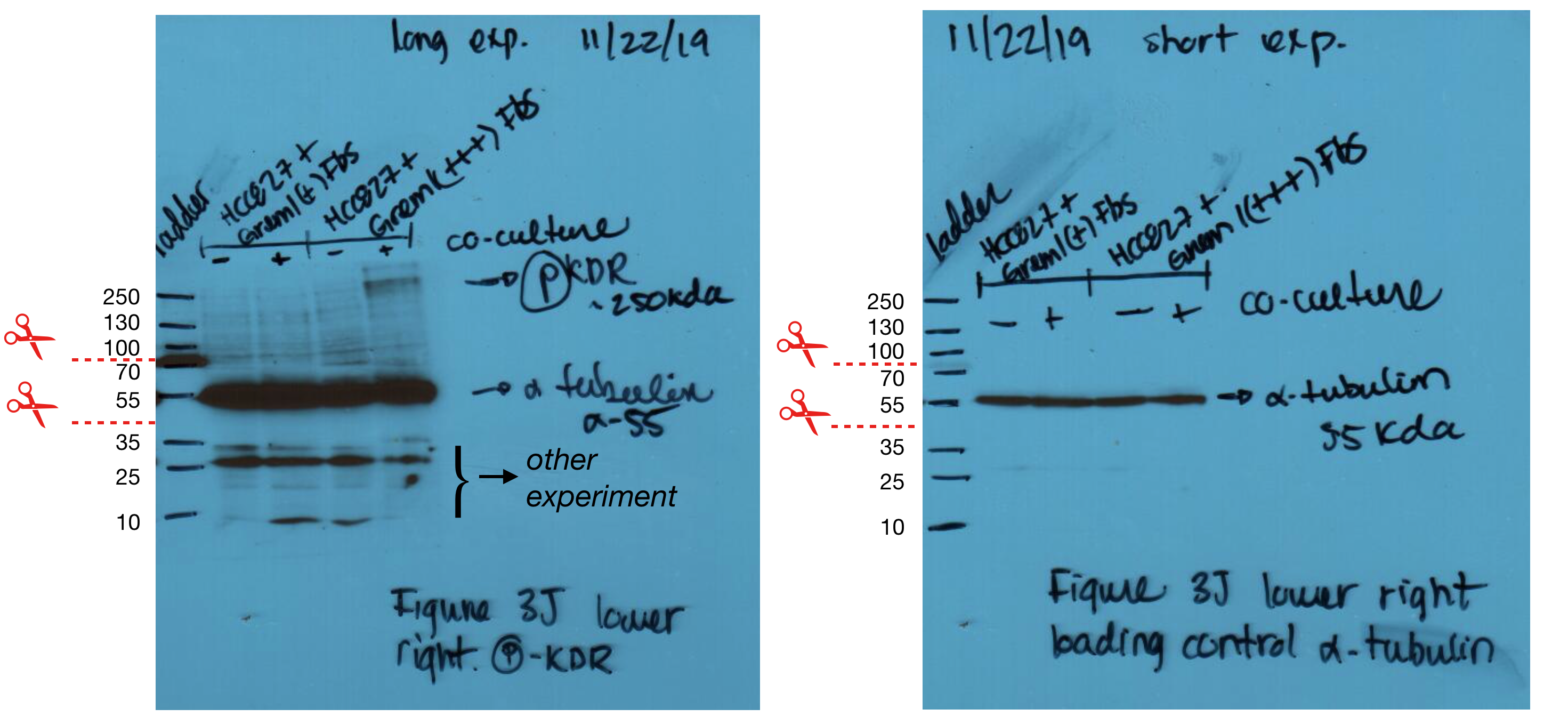 |
| 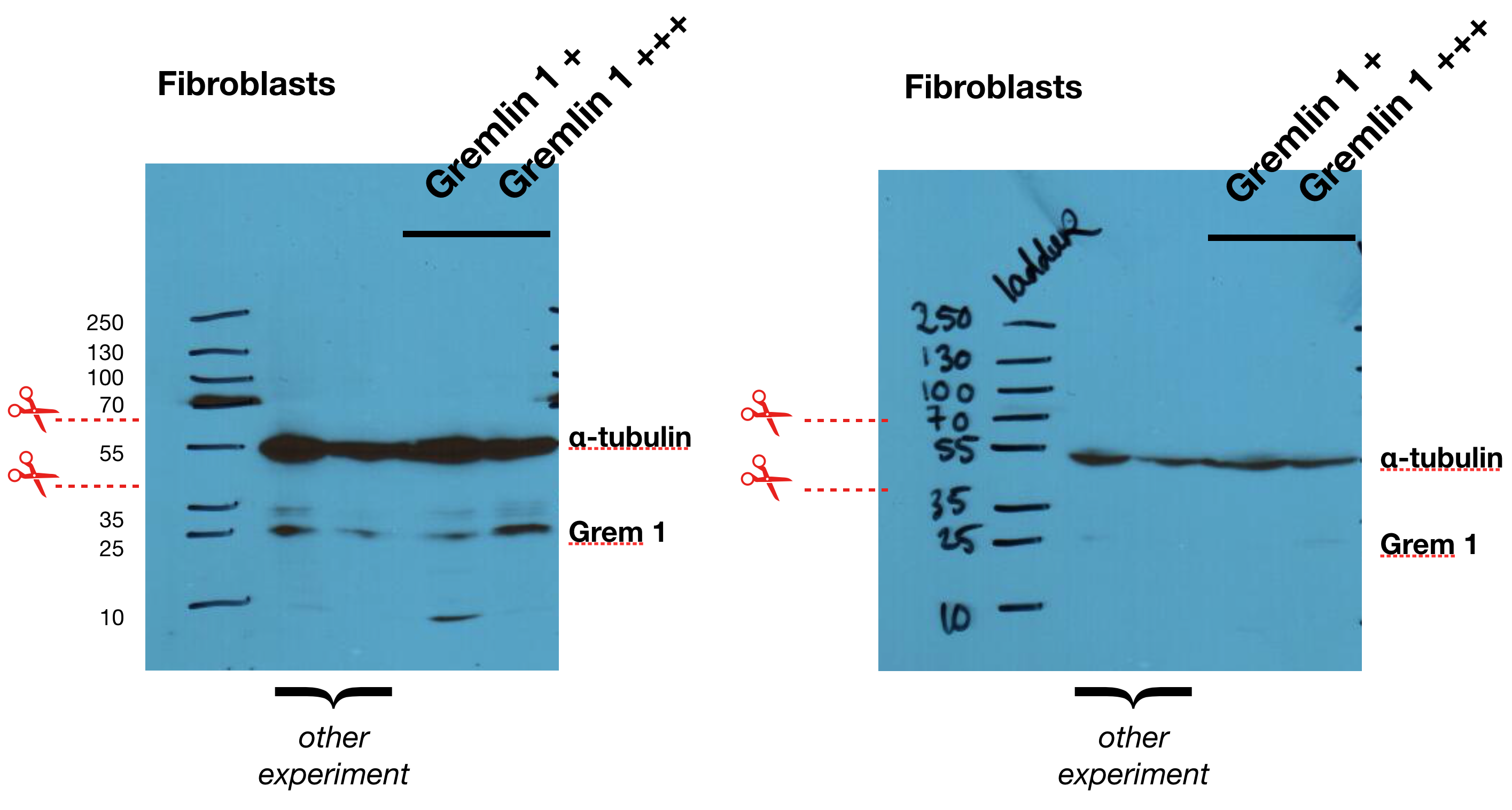 |
| Top row: (Left) KDR, exposure time 15 minutes; (Right) alpha-tubulin, exposure time 30 secs. Middle row: (Left) Phospho-KDR and alpha-tubulin, exposure time 15 mins; (Right) Phospho-KDR and alpha-tubulin, exposure time 1 min. Bottom row: Gremlin-1 and alpha-tubulin, exposure time 5 mins; (Right) Gremlin-1 and alpha-tubulin, exposure time 30 secs. |

**Figure S7**

TPSAB1 expression is 30-fold higher in mast cells relative to other immune cell types in the LM22 signature matrix from CIBERSORT. Bars show the Affymetrix MAS5 intensity levels, averaged across replicates for the indicated cell types.

**Figure S8: Mast cell counts across histologies, and across stages within adenocarcinoma and SCC.**

Mast cell counts across NSCLC histologies (top) on TMA; and across stage for adenocarcinoma (bottom left) and SCC (bottom right).

|  | |
| --- | --- |
|  |  |

**Figure S9: Mast cell tryptase and KI67 staining for a low-mast-cell infiltration adenocarcinoma**

**
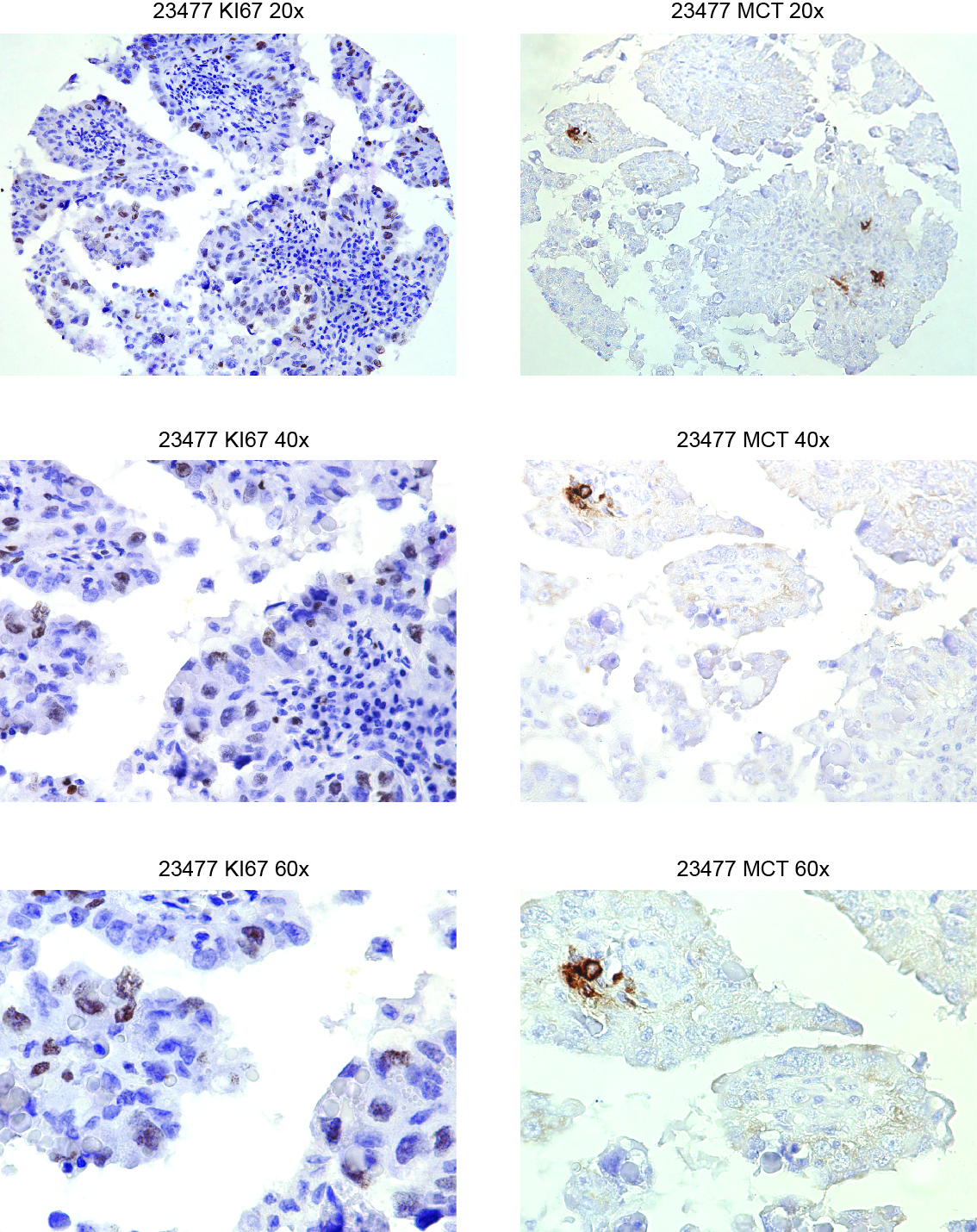
**

**Figure S10: Mast cell tryptase and KI67 staining for a high-mast-cell infiltration adenocarcinoma**

**
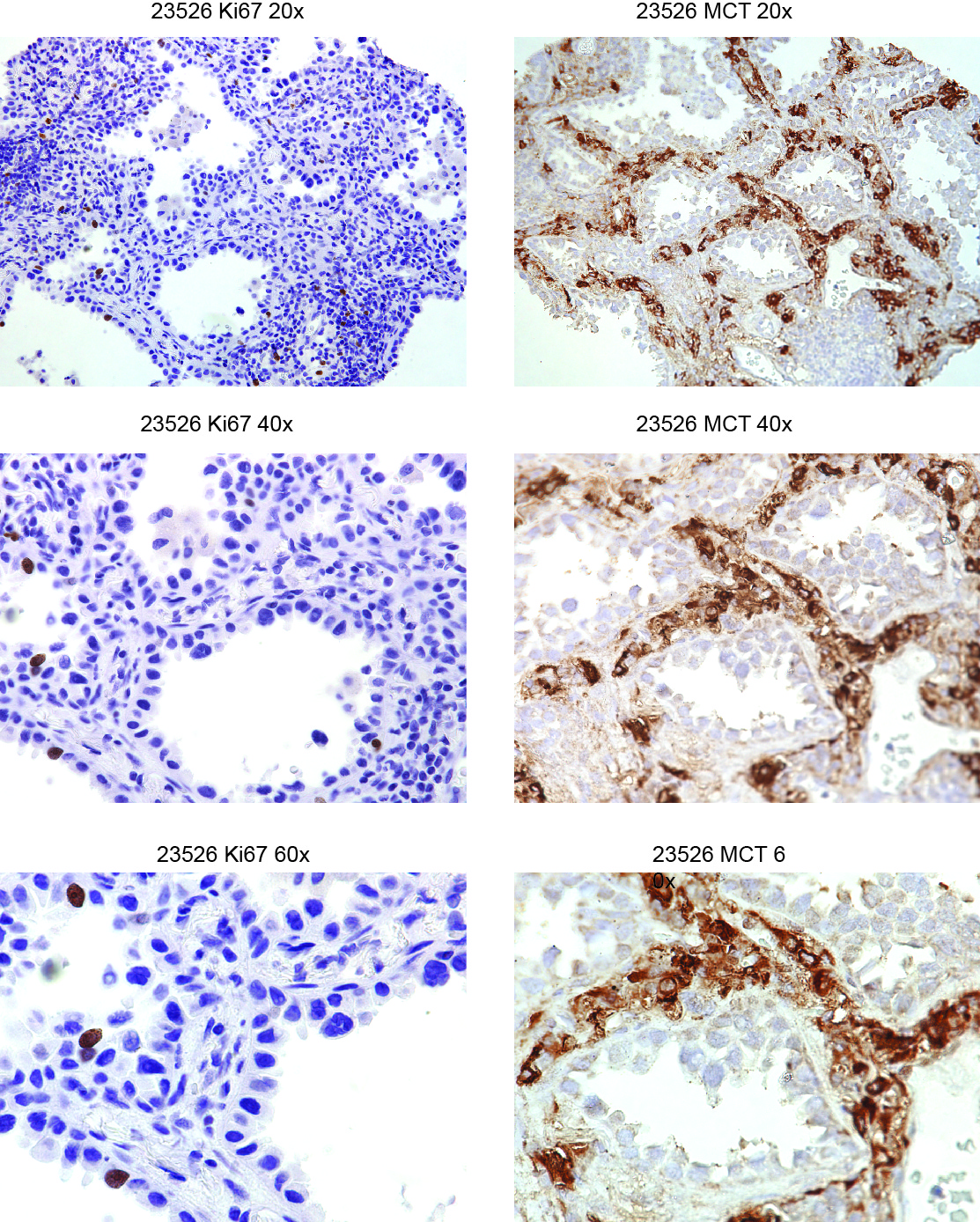
**

**Figure S11: Kaplan-Meier analysis of Mast cell association with overall survival across stages of NSCLC.**
