# Supplementary material for: A human lung tumor microenvironment interactome identifies clinically relevant cell-type cross-talk
Source: Genome Biol. 2020 May 7;21:107. doi: 10.1186/s13059-020-02019-x (PMC7206807; doi:10.1186/s13059-020-02019-x)
Supplement: Supplementary file 15 — Review history. [file 13059_2020_2019_MOESM15_ESM.docx]

Review History

**First round of review**

**Reviewer 1**

**Comments to author:**

This manuscript attempts to understand the complex interactions between cancer cells and stromal cells in lung cancer TME. Compared to traditional bulk RNA-seq profiling the authors sort select populations of stromal cells and cancer cells in 36 human tumors to conduct RNA-seq. They use the multicellular RNA-seq data to identify secreted ligands and cognate cell surface receptors receptors to computationally reconstruct cell-cell crosstalk between cells referred to as the lung tumor microenvironment interactome. Despite some strengths, potential weakeness pertaining to novelty, descriptive nature of investigation and lack of conceptual or mechanistic advances dampen overall enthusiasm for this work.

1) While the concept of using multicellular RNA-seq data to identify tumor-stroma crosstalk has the potential to dissect TME heterogeneity, this is not entirely novel concept and had been published previously in lung cancer (Choi et al. Cell Reports 2015). RNA-seq of individual cellular subsets in NSCLC and breast cancer has also been reported (Durrans et al. PlosOne 2015) (Allinen et al. Cancer Cell 2004).  Moreover, more recent studies have begun to evaluate cell-cell interactions a single cell level (Kumar et al. Cell Rep 2018; Trisoh et al. Science 2016). In light of these reports, the current study is incremental at best. It is also unclear why the authors have not put this study in the context of the existing literature.

2) There is concern with the approach used here to determine ligand receptor interaction in the TME. The authors assume that the ligands and the receptors are only produced by the cellular subsets they have profiled. There are other cellular fractions that were not profiled in this study that may be the source as well.

3) Merely identifying ligands receptors and downstream pathways is not sufficient to accurately predict interactions. The approach to identify activation of pathways downstream of the receptor may require analysis of phospho proteins and transcription factors.

4) The most important aspect of this study is the identification of potential cross talk between cell types in adenocarcinoma. However, this study falls short to achieving this goal. What is basically provided is a descriptive list of statistically significant ligands and receptors and the assumption that these interact in the TME.

5) The statement in line 42-47,  "Our results indicated potential for highly complex inter-population communication via ligand-receptor within the malignant compartment" is highly speculative and needs further characterization".


7) The authors stated that they would experimentally test if  GREM1 production by fibroblasts might induce a more aggressive malignant cell behavior in adenocarcinoma but not squamous cell carcinoma. However, the experimental approach is artificial as it used treatment of lung cancer cells with recombinant GREM1, and does not actually validate the fibroblast -tumor cell crosstalk.


8) Similarly, the finding that GREM1+ fibroblasts are in proximity of cancer cells is not indicative of a paracrine interaction. It would be important to show that either KDR is phosphorylated in the cancer cells or KDR downstream pathways are activated

9) The experimental validation of the cross talk is not sufficiently robust and no in vivo evidence in the context of the entire TME is presented.


Minor

Reference supporting that Gremlin binds VEGFR2 is not included.

**Reviewer 2**

**Comments to author:**

General comments

The manuscript by Gentles and colleagues describes a study on reconstructing cross-talk between tumor cells, endothelial cells, immune cells, and fibroblasts in the tumor microenvironment. The authors used sorted cells from primary non-small-cell lung tumors, carried out RNA-sequencing of the sorted individual cell types, and charted inter-population communication via ligand-receptor signaling. Using this lung tumor microenvironment interactome an unfavorable effect of Gremlin-1 (produced by fibroblasts) on proliferation of tumor cells was identified and validated. Finally, an association between infiltration of mast cells and less aggressive tumor cell behavior is described.

The study is highly innovative and provides novel biological insights into the complex heterocellular interaction in the tumor microenvironment. It was carried out using state-of-the art experimental and computational methods and could be of interest to a broader audience. Rather than using descriptive single-cell sequencing with incomplete transcriptome coverage, the authors decided to apply population sequencing of sorted individual cell types. Also careful analysis of the possible effects of the dissociation procedure on the transcriptomes was performed, further supporting the chosen experimental strategy. The finding that Gremlin-1 produced by fibroblasts stimulates proliferation is intriguing and stimulates further research in this direction. While overall favorable, there are several issues that need to be addressed in order to improve the manuscript.


Specific comments

1. The original CIBERSORT algorithm was developed based in microarray data. The authors should provide information how was the scaling to RNA-seq data performed.

2. One of the best repositories of curated receptors, ligands and their interactions is CellPhoneDB. The authors should integrate the LTMI data with this information to make the resource even more valuable. I also miss selection of patient subgroups on the web site which could be highly attractive (i.e. subgroups by histology, staging as in Suppl. Fig 7 and additionally subgroups by genetic features). Very likely the cell-cell networks will be different for different patient subgroups.

3. Is there an additional evidence on the prognostic relevance of mast cells? The authors could analyze publicly available datasets from bulk RNA-seq data and investigate association with angiogenesis and inflammation markers. This could further strengthen the finding and perhaps provide some hints on the causative relationship.

4. Second page of the Discussion, line 31: A reference is missing. The authors should also number the pages.

**Authors Response**

**Point-by-point responses to the reviewers’ comments:**

\# Reviewer 1:

This manuscript attempts to understand the complex interactions between cancer cells and stromal cells in lung cancer TME. Compared to traditional bulk RNA-seq profiling the authors sort select populations of stromal cells and cancer cells in 36 human tumors to conduct RNA-seq. They use the multicellular RNA-seq data to identify secreted ligands and cognate cell surface receptors receptors to computationally reconstruct cell-cell crosstalk between cells referred to as the lung tumor microenvironment interactome. Despite some strengths, potential weakeness pertaining to novelty, descriptive nature of investigation and lack of conceptual or mechanistic advances dampen overall enthusiasm for this work.

1) While the concept of using multicellular RNA-seq data to identify tumor-stroma crosstalk has the potential to dissect TME heterogeneity, this is not entirely novel concept and had been published previously in lung cancer (Choi et al. Cell Reports 2015). RNA-seq of individual cellular subsets in NSCLC and breast cancer has also been reported (Durrans et al. PlosOne 2015) (Allinen et al. Cancer Cell 2004).  Moreover, more recent studies have begun to evaluate cell-cell interactions a single cell level (Kumar et al. Cell Rep 2018; Trisoh et al. Science 2016). In light of these reports, the current study is incremental at best. It is also unclear why the authors have not put this study in the context of the existing literature.

2) There is concern with the approach used here to determine ligand receptor interaction in the TME. The authors assume that the ligands and the receptors are only produced by the cellular subsets they have profiled. There are other cellular fractions that were not profiled in this study that may be the source as well.

*We agree with the reviewer that this is a limitation of our study. In our paper, we attempt to allow for this by filtering for correlations of ligand expression with changes in gene expression of downstream genes in populations expressing the cognate receptor. We have commented more explicitly on this in the revised Discussion. We have also added to the LTMI website resource the capability to examine the expression of markers in NSCLC scRNA-seq data to permit users to assess this possibility. Nevertheless, even here not all cell types are represented in scRNA-seq, and there is always the possibility that the source of a ligand could be rare cells, or ones that are not captured due to technological limitations. Given the sparsity of scRNA-seq with currently available technologies, there is also high potential for missing ligand/receptor expression due to dropout. Hence we regard our study as complementary to bulk and single cell studies*.

3) Merely identifying ligands receptors and downstream pathways is not sufficient to accurately predict interactions. The approach to identify activation of pathways downstream of the receptor may require analysis of phospho proteins and transcription factors.

*Our resource is for generating hypotheses for subsequent follow up, not for making definitive causal inferences. No genomic resource or computational method can do the latter. The ultimate output of signaling pathways is whether their downstream pathways are transcriptionally activated. These would be the end point of phospho-signaling. A variety of validated computational approaches use the activity of transcriptional targets to infer the activity of an upstream regulatory protein, in lieu of directly measuring its phosphorylation state (for example, the MINDY, MIRANDA, and VIPER methods from the Califano laboratory). However, please see our response to points #7/8 where we describe additional experiments to evaluate the phosphorylation status of KDR in malignant cells when co-cultured with fibroblasts expressing different levels of GREM1.*

4) The most important aspect of this study is the identification of potential cross talk between cell types in adenocarcinoma. However, this study falls short to achieving this goal. What is basically provided is a descriptive list of statistically significant ligands and receptors and the assumption that these interact in the TME.

*As the reviewer notes, the aim of this resource is the identification of *potential* cross talk between cell types, not an exhaustive experimental validation of them. The latter is beyond the scope of this paper, and of most papers published in Genome Biology. Nowhere in our paper do we claim that these ligands and receptors definitely interact in the TME purely on the basis of the genomic data. However, we have been able to provide further experimental data to support a causative link between GREM1 expression and KDR phosphorylation in #7/8. We believe this highlights the utility of the LTMI as a resource for generating hypotheses regarding the TME.*

5) The statement in line 42-47,  "Our results indicated potential for highly complex inter-population communication via ligand-receptor within the malignant compartment" is highly speculative and needs further characterization".

*Again, we clearly state that these results indicate *potential* complex interactions – which they do. This is intrinsically descriptive/speculative, as are most of the studies mentioned in #1 including the seminal one by Tirosh et al.*

7) The authors stated that they would experimentally test if  GREM1 production by fibroblasts might induce a more aggressive malignant cell behavior in adenocarcinoma but not squamous cell carcinoma. However, the experimental approach is artificial as it used treatment of lung cancer cells with recombinant GREM1, and does not actually validate the fibroblast -tumor cell crosstalk.

*As indicated by staining for GREM1 mRNA in FFPE from primary tumor samples, fibroblasts are the only cells expressing this gene in the samples studied, and they are preferentially spatially located adjacent to malignant cells. However we concur that the treatment of cell lines with exogenous* *GREM1 is somewhat artificial. To better characterize the interaction between GREM1 from fibroblasts, and malignant cells, we have now performed additional experiments. We extracted cancer-associated fibroblasts (CAFs) from new primary human lung tumor samples and characterized their expression of GREM1. We then co-cultured low- and high-GREM1 expressing CAFs with lung cancer cell lines and evaluated the effect on the proliferation of the latter via flow cytometry using antibodies against KI67 and e-Cadherin (the latter to guard against cross-contamination). We found that co-culturing with high-GREM1 CAFs resulted in higher numbers of proliferating malignant cells. As suggested by the reviewer in #8, we also evaluated the phosphorylation of KDR in malignant cells. Indeed, co-culturing with GREM1+ CAFs resulted in KDR phosphorylation. Taken together these results are consistent with GREM1 from CAFs engaging KDR in malignant cells, resulting in its phosphorylation and resulting increases in their proliferation. We thank the Reviewer for this suggested additional validation, which we believe significantly improves confidence that the LTMI provides a useful way for generating hypotheses regarding the NSCLC TME.*


8) Similarly, the finding that GREM1+ fibroblasts are in proximity of cancer cells is not indicative of a paracrine interaction. It would be important to show that either KDR is phosphorylated in the cancer cells or KDR downstream pathways are activated.

*Please see our detailed response to #7.*

9) The experimental validation of the cross talk is not sufficiently robust and no in vivo evidence in the context of the entire TME is presented.

*In vivo evidence would require clinical trials, or evaluation in mouse models which do not accurately reproduce the TME of human cancers. We hope that the Reviewer will find the new data provided in #7 to be more compelling.*

Minor

Reference supporting that Gremlin binds VEGFR2 is not included.

*We have added the appropriate reference (Mitala et al Blood 2010), as well as our own new experimental data described in #7.*

\# Reviewer 2

General comments

The manuscript by Gentles and colleagues describes a study on reconstructing cross-talk between tumor cells, endothelial cells, immune cells, and fibroblasts in the tumor microenvironment. The authors used sorted cells from primary non-small-cell lung tumors, carried out RNA-sequencing of the sorted individual cell types, and charted inter-population communication via ligand-receptor signaling. Using this lung tumor microenvironment interactome an unfavorable effect of Gremlin-1 (produced by fibroblasts) on proliferation of tumor cells was identified and validated. Finally, an association between infiltration of mast cells and less aggressive tumor cell behavior is described.

The study is highly innovative and provides novel biological insights into the complex heterocellular interaction in the tumor microenvironment. It was carried out using state-of-the art experimental and computational methods and could be of interest to a broader audience. Rather than using descriptive single-cell sequencing with incomplete transcriptome coverage, the authors decided to apply population sequencing of sorted individual cell types. Also careful analysis of the possible effects of the dissociation procedure on the transcriptomes was performed, further supporting the chosen experimental strategy. The finding that Gremlin-1 produced by fibroblasts stimulates proliferation is intriguing and stimulates further research in this direction. While overall favorable, there are several issues that need to be addressed in order to improve the manuscript.

*We thank the reviewer for their comments which note the originality of the approach and the advantages relative to single cell RNA-seq.*

Specific comments

1. The original CIBERSORT algorithm was developed based in microarray data. The authors should provide information how was the scaling to RNA-seq data performed.

*A new version of CIBERSORT was recently published which extends the methodology to RNA-seq by using a novel batch correction method (Newman et al. Nature Biotech 2019). We have added a reference to this paper, as the new version of CIBERSORT was not publicly available when we originally submitted this work. We also note that notwithstanding the new paper, numerous groups have applied the original version of CIBERSORT to RNA-seq data and validated their findings.*

2. One of the best repositories of curated receptors, ligands and their interactions is CellPhoneDB. The authors should integrate the LTMI data with this information to make the resource even more valuable. I also miss selection of patient subgroups on the web site which could be highly attractive (i.e. subgroups by histology, staging as in Suppl. Fig 7 and additionally subgroups by genetic features). Very likely the cell-cell networks will be different for different patient subgroups.

*We thank the reviewer for bringing the new CellPhoneDB to our attention. We have added the possibility for selecting ligand-receptor pairs from this resource to the LTMI website. We considered merging it with the known lists that we used originally from FANTOM 5, but felt this could be confusing to readers. It will also be easier to update this aspect of the website along with future versions of CellPhoneDB, while leaving our original set of ligand-receptor pairs intact so as not to result in a continually shifting set of results which makes version tracking troublesome. Regarding selection by patient subgroups, we completely agree that this would be a valuable feature. Unfortunately, we are constrained by the cohort size. Particularly in the case of specific genetic features, there would be too few samples for obtaining cross-correlations from the expression data. We have added this limitation to the discussion, which limits us to features of the biology that are shared across subgroups.*

3. Is there an additional evidence on the prognostic relevance of mast cells? The authors could analyze publicly available datasets from bulk RNA-seq data and investigate association with angiogenesis and inflammation markers. This could further strengthen the finding and perhaps provide some hints on the causative relationship.

*Interestingly, the evidence for prognostic relevance for mast cells in lung cancer is very contradictory. We had inadvertently missed including the relevant references in the previous version of the manuscript and have corrected this. On the one hand some studies (e.g. Welsh et al) have indicated a favorable association, whereas others (e.g. Takanami et al) suggest an unfavorable association through promotion of angiogenesis. Concordant with this, we find that analysis of bulk NSCLC datasets via our PRECOG resource indicates a significant adverse association between expression of the angiogenesis markers VEGFA and VEGFC (but not VEGFB) with worse outcome in adenocarcinoma. The direction of this relationship is also present in SCC, but does not reach statistical significance, possibly because of the much smaller datasets available for this histology. We have expanded on the discussion to highlight some of these points. Whether our validation analysis via TMA staining, which is the largest cohort we are aware of, will resolve this remains to be seen. It seems likely that a more detailed understanding of the spatial location of mast cells is necessary, together with deeper knowledge of their functional diversity.*

4. Second page of the Discussion, line 31: A reference is missing. The authors should also number the pages.

*We have added the missing reference, and numbered the pages as requested.*

**Second round of review**

**Reviewer 2**

The authors addressed all issues raised in the previous review satisfactorily. Specifically, the integration of CellPhoneDB and the discussion on mast cells removed the remaining concerns. Both, the methodological approach of using population sequencing of sorted individual cell types as well as the presented results and the website are of interest to a broader audience.


Responses to Referee 1

The concerns of Referee 1 have been addressed appropriately as follows.

1. Previously published papers do address the question, however, they have major limitations as highlighted by the authors in the revised version on page 14. The limitations include the use of mouse models rather than human samples, small number of samples, and lack of validation.

2. Clearly, one limitation of the study is that other cellular fractions that were not profiled might also produce ligand and receptors and therefore could induce noise to the results. Since the major fractions were profiled, it is unlikely that the noise generated by minor or rare fractions will have considerable impact on the results. Hence, the presented study is complementary to other studies using bulk or scRNA-seq and provides novel insights.

3. Analyses of phosphoproteins or transcription factors could indeed provide important information. However, due to the sensitivity of the mass spec instruments, comprehensive phosphoproteomic analysis is not feasible with biopsies and only a proxy using RNA data can be generated. The authors pointed also to the methods that can be used for this type of analysis. A minor suggestion is to include part of the response and the references in the discussion section.

4. and 5. As the authors responded, an exhaustive experimental validation of the identified cross talk is beyond the scope of a single study.

7. The inclusion of additional experiments on GREM1 and the provision of compelling evidence definitely strengthens the manuscript.

9. Further experimental validation could be a subject of another study.

In summary, the authors addressed also the concerns of Referee 1 and thereby considerably improved that manuscript.
